# Mechanical Damage Assessment for Pneumatic Control Valves Based on a Statistical Reliability Model

**DOI:** 10.3390/s21103307

**Published:** 2021-05-11

**Authors:** Nirbhay Mathur, Vijanth Sagayan Asirvadam, Azrina Abd Aziz

**Affiliations:** 1 Center for System Engineering, Institute of Autonomous Systems, Univiersiti Teknologi PETRONAS, Seri Iskander 32610, Perak, Malaysia; nirbhay.mathur@utp.edu.my; 2 Department of Electrical and Electronics Engineering, Univiersiti Teknologi PETRONAS, Seri Iskander 32610, Perak, Malaysia; azrina_aaziz@utp.edu.my

**Keywords:** fault detection, control valve, reliability, visualization

## Abstract

A reliability assessment is an important tool used for processing plants, since the facility consists of many loops and instruments attached and operated based on other availability; thus, a statistical model is needed to visualize the reliability of its operation. The paper focuses on the reliability assessment and prediction based on the existing statistical models, such as normal, log-normal, exponential, and Weibull distribution. This paper evaluates and visualizes the statistical reliability models optimized using MLE and considers the failure mode caused during a simulated process control operation. We simulated the failure of the control valve caused by stiction running with various flow rates using a pilot plant, which depicted the Weibull distribution as the best model to estimate the simulated process failure.

## 1. Introduction

Processing plants consist of a large number of components. As industries have already moved into the era of digitization, these automatized operations need to be reliable. Thus, it is necessary to look into all parameters which affect the cost of overall operation; hence, assessments of reliability are essential for any plant. Each machine/plant consists of a large number of sub-systems and each sub-system has many large components attached with each other.

Reliability analysis can be performed by both qualitative and quantitative measurements, as [1] reliability is the probability of equipment staying operational without failing for a given time interval [2]. The reliability of the plant/machine can be found in regard to normal operating conditions or during high production or scheduled outages [3].

Reliability studies have been carried out by many researchers, and this study looked into control valve reliability in the processing plant. This paper contributes two major aspects: failure analysis of valves due to stiction and the visualization of failures based on the reliability models. In processing plants, control valves are considered as the actuators for process regulation. Performance and reliability play significant roles in keeping the process steady and accurate [4]. On that note, it has been said that about 30% of valve nonlinearity problems occur due to stiction, deadbanding and hysteresis [5]. As the nonlinearity in the process plants increases, the oscillation for the control valve will accelerate towards degradation, which may increase the operational costs and in long term produce lower output [6] also will cause major losses to the plant [7,8].

Valve stiction, also called static friction, is considered the one of the sources of process failure, and 0.5% stiction can cause a large disturbance to a system by producing an uncontrolled output which can harm the life of the valve itself [9].

In order to understand the concept of reliability, recently we discussed the life cycle of control valves and reliability estimation [10]. W. Y. Jain conducted a reliability assessment on a solenoid valve for a high-speed train braking system [11]. The traditional reliability assessment method is only operation to use when given a large sample size and a complete list of failure data. Tiefeng Zhu [12] proposed reliability estimation for a two-parameter Weibull distribution for block censoring, which is compared with the Bayes method to predict reliability measurement. Darja did work on different levels of reliability in hazardous environments, where the systems are kept in good working condition continuously [13].

The present study proposes a visualization technique and justifies the right statistical model for process failure. The reliability assessments are the major factor to keep processing plants in working condition, and it is hard to implement all existing models to estimate the reliability prediction for all systems in a processing plant. This paper is divided into four major sections. Section 1 addresses the introduction of reliability and control valves. In Section 2, the reliability assessment is discussed, and in Section 3, data collection based on simulation of a single-tank pilot plant is explained along with a statistical reliability model. Section 4 discusses the results, and Section 5 concludes and discusses the findings of the paper.

## 2. Reliability Assessment and Its Components

This section discusses the assessment of the reliability and the main components of reliability and their usages to estimate failure.

### 2.1. Reliability Assessment

Reliability assessment is a term that relates to evaluating the capability of a given process in operation to obtain good performance over service time. In order to maintain long life and low-cost maintenance, it is necessary to design a reliability assessment properly [11]. The other components of reliability assessments are availability and maintainability, which together form RAM measurement.

The reliability failure of a system (F(t)) can be estimated by considering the cdf and probability of failure (f(t)). The failure distribution λ(t) can be expressed as follows:

(1) F(t)=1−R(t)

(2) f(t)=dF(t)/dt

(3) λ(t)=f(t)/R(t)

(4) h(t)=1/λ(t)

In mean time to failure (MTTF) plays an important role in achieving reliability equations. The MTTF can be calculated as follows:

(5) MTTF=(Totalhoursofoperation)/(Totalcomponentinuse)

$$MTTF=E\left(t\right)=\int_{0}^{\infty }tf\left(t\right)dt$$

### 2.2. Bath Tub Curve

**Figure 1:**
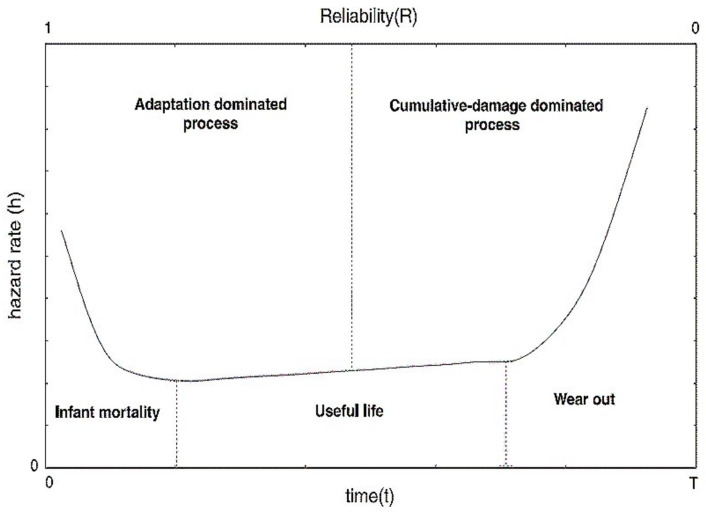
The bathtub curve with three phases.

The bathtub curve is a graphical representation of failure rate over time, and the name was given as the plot resembles a bathtub in shape. This type of curve represents the lifetime of the product/component and describes the failure pattern according to the aging of the product based on time. This curve is divided into three main cycle phases. The first is called the infant mortality region when the system is newly introduced and has a high failure rate. The second is known as the constant failure rate when the product is stable with few failures. The third is known as the wear-out region, when the failure rate is significantly increased. The bathtub curve is shown in Figure 1 and is also depicted in other sections to visualize component lifetime clearly.

## 3. System Setup

This section discusses how data are generated and the methodology used for measuring and visualizing failure using different statistical models.

### 3.1. Experimental Setup

**Figure 2:**
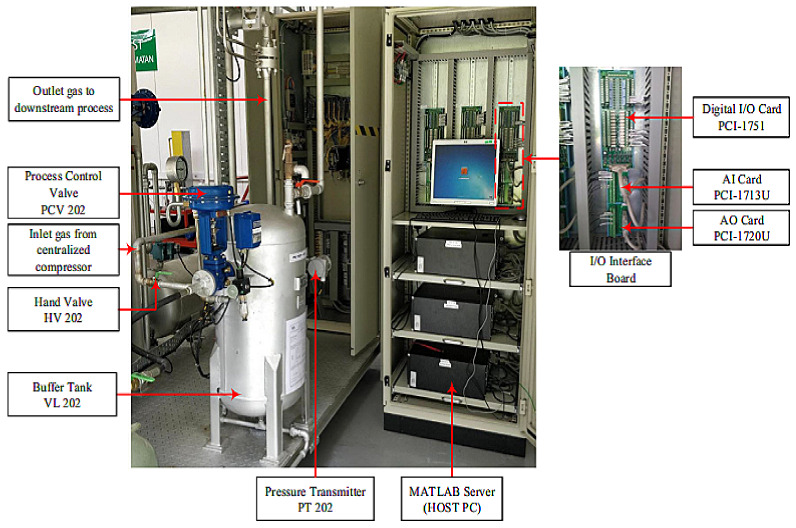
Single-tank pilot plant setup for data collection.

The experiment was conducted in a single-tank pilot plant, as shown in Figure 2. It consisted of a pressure tank, VL 202, connected to a pressure transmitter through hand valve HV 202, which supplied gas input continuously; and in order to manipulate the input, a pressure control valve PCV 202 was connected. A pneumatic control valve PCV 202 regulated the gas according to the setpoint by adjusting gate valve opening from 0 to 100%. The tank’s pressure was monitored and measured by the pressure transmitter PT 202 and the internal pressure controller PIC 202.

**Figure 3:**
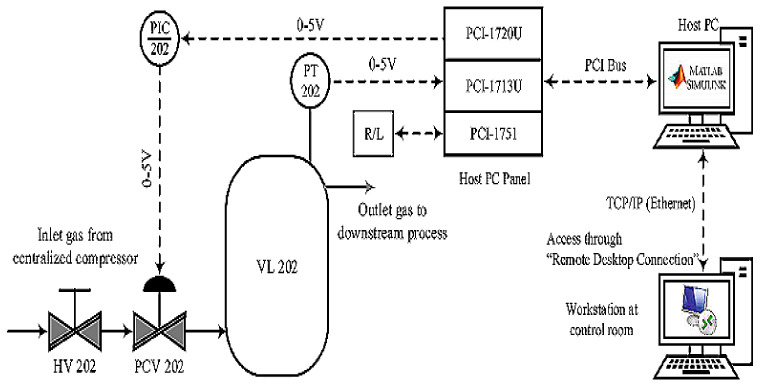
A block diagram of the single-tank system simulated in MATLAB for extracting failure data.

**Figure 4:**
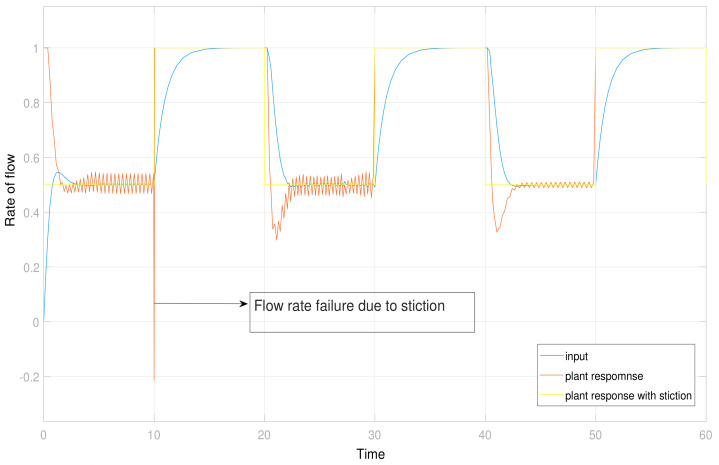
A control valve operation plot to see the behavior of stiction in comparison of set point.

All components were connected with the host PC using MATLAB/SIMULINK software, and peripherals such as PCI cards 1713U, 1720, and 1751 were used to connect to the host PC. The pressure transmitter PT 202 was accessed through PCI 1713U, which is a 32-channel analog universal PCI card. The control signal was connected with a 4-channel isolated D/A output card, which was later linked with PCV 202 through PCI 1720. In order to operate the process plant remotely or locally, a 48-bit I/O counter card (PCI 1751) was used to communicate with the process plant and to collect the data into the MATLAB application offline or in real time. A schematic diagram of the single-tank pilot plant’s simulation incidence is shown in Figure 3, whereas Figure 4 shows the output in the form of rate of flow.

### 3.2. Data Collection

Significant failures occur in processing plants due to control valve failures, which may create nonlinearity in the system. One of the main valve type failures is known as the stiction problem [14,15], which may be depicted as a periodic random impulse in the rate of flow.

**Figure 5:**
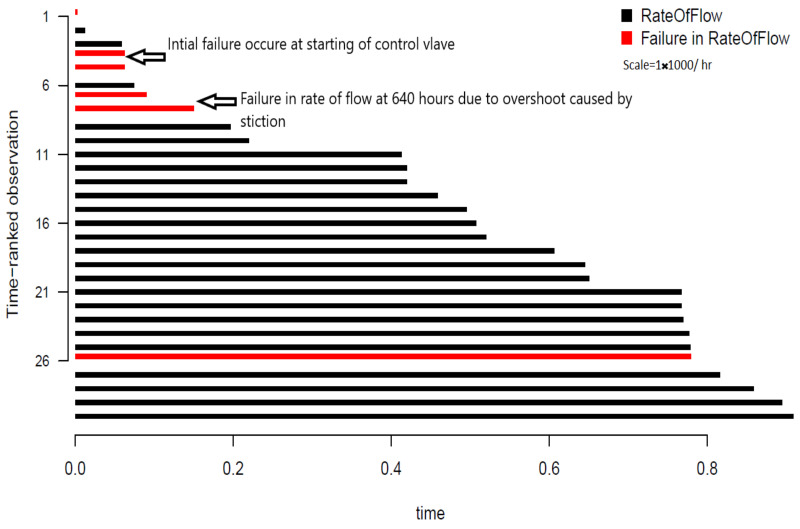
Censored failure data collected from simulation.

In order to categorize failure, data transformation is performed using censored interval data which can be derived as

(6) $$\hat{F}\left(t_{i}\right)=\frac{\left(\mathrm{Number}\;\mathrm{of}\;\mathrm{failure}\;\mathrm{up}\;\mathrm{to}\;\mathrm{time}\;\left(t_{i}\right)\right)}{n}=\frac{\sum_{j=1}^{i}\left(d_{j}\right)}{n}$$

where *n* is the total sample size, $\left(d_{i}\right)$ denotes the number of failures recorded due to overshooting for the ith interval of time $\left(t_{i-n},t_{i}\right)$. Hence, the non-parametric estimation is denoted by the overtime interval, as shown in Figure 5. The figure depicts the rate of flow under time ranked observation, in which the real value of the rate of flow is replaced by an ascending order ranking system. This form of data visualization enables visualization of failure due to a sudden overshoot in the rate of flow interval of time, which is represented by the red bar in Figure 5. The other red bars represent early stage and end of life failure. More explanation of failure data is discussed in the following sections.

### 3.3. Reliability Prediction

As said earlier, this paper discusses several statistical reliability models for predicting time to failure (TTF) for the control valve. The four models used to measure the reliability of the system or experiment are discussed next.

#### 3.3.1. Weibull Distribution

The Weibull distribution was initially used to model the breaking strength of the component and for predicting the life cycle analysis for the components or instruments. The two parameters of the Weibull distribution are also widely used to calculate the cumulative density function (cdf), which is written as

(7) $$Pr\left(T\leq t;\eta ,\beta \right)=1-exp[-\left({\frac{t}{\eta }}^{\beta }\right)],t>0$$

where β>0 represents the shape parameters and η>0 represents the scale parameter. Let us assume that if *T* has a Weibull distribution, then Y=log(T)∼sev(μ,σ), where sev is the survival distribution, σ=1/β is the scale parameter and μ=log(η) is the location parameter. As per the assumptions, *T* has a Weibull distribution, which can be represented by T∼weib(μ,σ) [16]. Now the Weibull cdf, pdf and λ(t) can be written as:

(8) $$F\left(t;\mu ,\sigma \right)=\Phi_{sev}\left[\frac{log(t)-\mu }{\sigma }\right],$$

(9) $$f\left(t;\mu ,\sigma \right)=\frac{1}{\sigma t}\phi_{ssev}\left[\frac{log(t)-\mu }{\sigma }\right]=\frac{\beta }{\eta }{\left(\frac{t}{\eta }\right)}^{\beta -1}exp\left[-{\left(\frac{t}{\eta }\right)}^{\beta }\right]$$

$$\lambda \left(t\right)=\frac{f(t)}{F(t)}$$

#### 3.3.2. Normal Distribution

The probability of failure using normal distribution can be derived as follows:

(10) $$F\left(y;\mu ,\sigma \right)=\Phi_{nor}\left(\frac{y-\mu }{\sigma }\right)$$

(11) $$f\left(y;\mu ,\sigma \right)=\frac{1}{\sigma }\phi_{nor}\left(\frac{y-\mu }{\sigma }\right),-\infty <y<\infty ,$$

where $\phi_{nor}=\left(1\sqrt{2\pi }\right)exp\left(-z^{2}/2\right)$ and $\Phi_{nor}\left(z\right)=\int_{-\infty }^{z}\Phi_{nor}\left(w\right)dw$ are respectively the pdf and cdf for standardized nor(μ=0,σ=1) distribution.

The normal distribution is well known for its simplicity and center limit application [16]. However, when it comes to reliability analysis, the normal distribution is less used because its hazard rate h(t) calculation is valid until the median of the data. The normal distribution is useful only with some limited data set with limited life cycle data in which the following condition is true (i.e., μ>0) and coefficient variation (σ/μ).

#### 3.3.3. Exponential Distribution

The failure rate of exponential distribution can be denoted by *T*∼exp(θ,γ) and the two-parameter exponential distribution can be expressed for cdf and pdf functions as follows:

(12) $$F\left(t;\theta ,\gamma \right)=1-exp\left(-\frac{t-\gamma }{\theta }\right)$$

(13) $$f\left(t;\theta ,\gamma \right)=\frac{1}{\theta }exp\left(-\frac{t-\gamma }{\theta }\right)$$

θ>0 is considered a scale parameter, and γ is a location parameter. As per [16], an exponential distribution is most commonly used for reliability measurements in electronic components. Simultaneously, the exponential distribution is not commonly used for data sets with component failure due to defects.

#### 3.3.4. Lognormal Distribution

The final comparison was made with the lognormal distribution, which can be denoted by *T*∼lognor(μ,σ). The cdf and pdf for the lognormal distribution can be derived as

(14) $$F\left(t;\mu ,\sigma \right)=\Phi_{nor}\left[\frac{log(t)-\mu }{\sigma }\right]$$

and

(15) $$f\left(t;\mu ,\sigma \right)=\frac{1}{\sigma t}\phi_{nor}\left[\frac{log(t)-\mu }{\sigma }\right],t>0$$

where $\phi_{nor}$ and $\Phi_{nor}$ are the pdf and cdf for a standard normal distribution. This distribution is used in the literature for estimating failure via degradation processes [17,18].

## 4. Simulation Results

**Figure 6:**
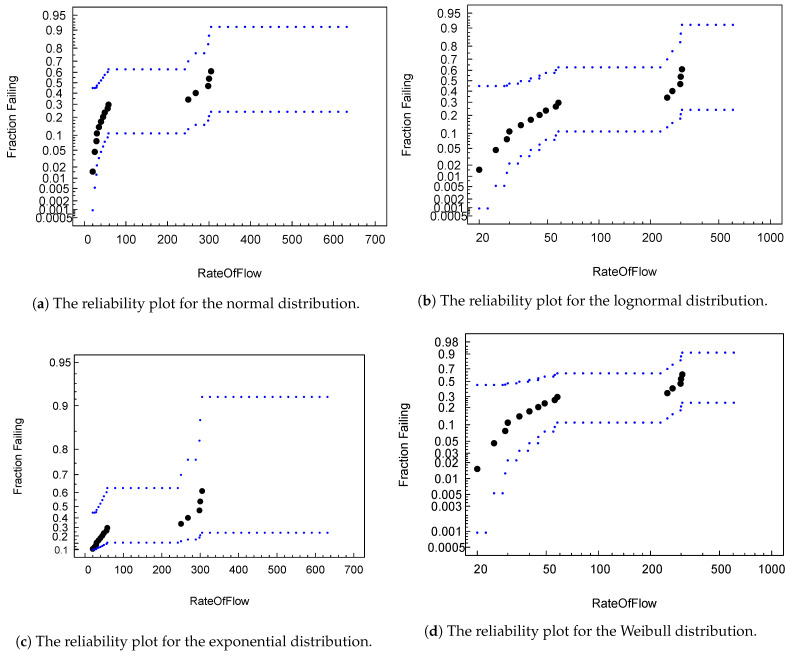
A plot to visualize the different distribution fits with failure data.

Data obtained with failure elements were modeled to fit the statistical reliability models mentioned in the previous section. Figure 6 shows four plots representing four reliability models by populating data with various flow rates. Though the figures look similar, the Weibull distribution (Figure 6d) provides the best confidence band fitting in comparison with other models.

Table 1 shows that when the rate of flow from the pneumatic control valve rises, the failure rate $\hat{F}$ tends to rise too, which may lead to higher chances of valve failure.

Table 2 indicates the mean times to failure (MTTF) for the different reliability models that estimate the system’s time to failure. Table 2’s results can be compared with Figure 5, which indicates time ranked observation in the presence of a process failure. By referring to Figure 5, the failure rate obtained through MTTF for a normal distribution represents early stage failure due to the initial phase of process plant operations. The Weibull distribution depicts actual midterm failure due to stiction, whereas log normal and exponential distributions represent overloaded conditions at the ends of operations.

Table 3 shows the parameters of each statistical reliability model optimized by using MLE.

## 5. Conclusions and Discussion

The paper aimed to visualizes faults and present a reliability assessment of pneumatic valve failure, which is often used in processing plants. The data were collected from a single-tank pilot plant system in which the failure of a pneumatic control valve was simulated for different rates of flow. Data with process failure were transformed in order to time rank them for categorizing process failures. These censored time rank failure observations could relate to the MTTF obtained from the four statistical reliability models. Combining reliability assessments and visualizations may help the systems engineers when it is necessary to perform maintenance in order to keep processing plants in working condition. Future work will look into developing a smart dashboard which incorporates any type of instrument or industry subsystem in order to guess the working behavior and its reliability by fitting it with a set of statistics-based reliability models.

## Figures and Tables

**Table 1:** Failure rate calculated based on different stages of rate of flow for pilot plot data.

Rate of Flow	$\hat{F}$	Std. Error	MLE 95% CI	
			95% Lower	95% Upper
20	0.04945167	0.03025560	0.01471353	0.1593042
50	0.10078031	0.04481346	0.04147054	0.2338855
100	0.16959199	0.05828572	0.08482773	0.3226762
200	0.27755249	0.07851267	0.08482773	0.3226762
500	0.49386968	0.13000931	0.27755502	0.7598024
1000	0.69617176	0.16954764	0.37852058	0.9493920

**Table 2:** MTTF obtained from different statistical reliability models.

Model	MTTF	Model 95% CI	
		95% Lower	95% Upper
Weibull Distribution	652.6 h	106.5	4000
Normal	253.1 h	132.3	373.9
Lognormal	8952.00 h	115.5	694,103.00
Exponential	25,450.00 h	9552	67,809.00

**Table 3:** Statistical model parameters optimized using MLE.

Model Parameters	MLE	Stand Err	Model 95% CI	
			95% Lower	95% Upper
Weibull μ	6.6012	0.9328	4.773	8.4295
Weibull σ	0.4108	0.1584	0.193	0.8745
Weibull η	736.0062	686.5469	118.272	4580.1809
Weibull β	2.4341	0.9383	1.143	5.1814
Normal μ	253.12	61.64	132.30	373.9
Normal σ	66.14	21.80	34.66	126.2
Lognormal μ	8.097	1.4848	5.1867	11.007
Lognormal σ	1.416	0.5233	0.6864	2.922
Exponential μ	10.14	0.5	9.164	11.12
Exponential σ	1.00	0.0	1.000	1.00

## Data Availability

Data for the pilot plant are available and can be retrieved from the author’s GitHub account: https://github.com/mathur01/ReliabilityData, accessed on 23 February 2021.

## Abbreviations

The following abbreviations are used in this manuscript:

RAM	Reliability Availability and Maintainability
MTTF	Mean Time To Fail
TTF	Total Time to Fail
weib	Weibull Distribution
exp	Exponential Distribution
lognor	Log Normal Distribution
cdf	Cumulative Density Function
pdf	Probability Density Function
f(t)	pdf of random variable
F(t)	cdf for a system
h(t)	Hazard Function
λ(t)	failure rate
MLE	Maximum Likelihood Estimation
Std	Standard Error
CI	Confidence Intervals
Pr	Probability
nor	Normal
η	eta, Weibull scale parameter
β	beta, Weibull distribution shape parameter
sev	Survival, smallest extreme value distribution
ssev	indicates a standard smallest extreme value distribution
Φ	cdf for a standardized location-scale distribution
ϕ	pdf for a standardized location-scale distribution
σ	sigma, scale parameter for a location-scale distribution
μ	mu, location parameter of a location-scale distribution
γ	gamma, threshold parameter of the distribution function of T
*y*	unrestricted random variable or a dummy variable
*z*	standard random variable
